# Causal association between circulating inflammatory markers and sciatica development: a Mendelian randomization study

**DOI:** 10.3389/fneur.2024.1380719

**Published:** 2024-07-02

**Authors:** Yang Wu, Yi Lin, Mengpei Zhang, Ke He, Guihua Tian

**Affiliations:** ^1^Dongzhimen Hospital, Beijing University of Chinese Medicine, Beijing, China; ^2^School of Computer Science and Technology, Beijing Institute of Technology, Beijing, China

**Keywords:** sciatica, inflammation, circulating inflammatory markers, Mendelian randomization, genetic analysis, epidemiology, GWAS

## Abstract

**Background:**

This research explores the causal association between circulating inflammatory markers and the development of sciatica, a common and debilitating condition. While previous studies have indicated that inflammation may be a factor in sciatica, but a thorough genetic investigation to determine a cause-and-effect relationship has not yet been carried out. Gaining insight into these interactions may uncover novel treatment targets.

**Methods:**

We utilized data from the OpenGWAS database, incorporating a large European cohort of 484,598 individuals, including 4,549 sciatica patients. Our study focused on 91 distinct circulating inflammatory markers. Genetic variations were employed as instrumental variables (IVs) for these markers. The analysis was conducted using inverse variance weighting (IVW) as the primary method, supplemented by weighted median-based estimation. Validation of the findings was conducted by sensitivity studies, utilizing the R software for statistical computations.

**Results:**

The analysis revealed that 52 out of the 91 inflammatory markers studied showed a significant causal association with the risk of developing sciatica. Key markers like CCL2, monocyte chemotactic protein-4, and protein S100-A12 demonstrated a positive correlation. In addition, there was no heterogeneity or horizontal pleiotropy in these results. Interestingly, a reverse Mendelian randomization analysis also indicated potential causative effects of sciatica on certain inflammatory markers, notably Fms-related tyrosine kinase 3 ligands.

**Discussion:**

The study provides robust evidence linking specific circulating inflammatory markers with the risk of sciatica, highlighting the role of inflammation in its pathogenesis. These findings could inform future research into targeted treatments and enhance our understanding of the biological mechanisms underlying sciatica.

## 1 Introduction

Sciatica refers to pain caused by inflammation or compression of the sciatic nerve, which is the largest and longest nerve in the body (1, 2). It originates in the lower back and runs through the buttocks and down the lower limbs (3). Sciatica is a common and debilitating condition, with a lifetime prevalence estimated at 13%–40% (1). It exerts substantial burdens due to chronic pain, missed work, reduced productivity, and social limitations (4). Currently, treatment options are limited for many patients. A better understanding of the pathological processes underlying sciatica is critical to inform improved management strategies.

While the exact mechanisms driving sciatica remain unclear, accumulating evidence suggests inflammation may play a vital role (5, 6). Several inflammatory molecules including cytokines and chemokines have been found to be elevated in serum, plasma, and cerebrospinal fluid of sciatica patients compared to healthy people, such as TNF-α, IL-6, IL-8, and IL-17 (7). Additionally, clinicians commonly use anti-inflammatory medications like NSAIDs to treat sciatica, providing indirect evidence that targeting inflammation may be beneficial (8). However, most previous studies have been limited to observational associations between inflammatory markers and sciatica. Establishing causal relationships is vital to understand if inflammation actively contributes to sciatic nerve pathology or merely represents an epiphenomenon.

Circulating inflammatory markers refer to a diverse array of proteins and signaling molecules present in the blood or other bodily fluids that are involved in regulating inflammatory and immune responses (9). These include cytokines, chemokines, acute phase proteins, proteases, and cellular markers released by activated immune cells (10). Cytokines like interleukins (e.g., IL-6, IL-8) and tumor necrosis factor-alpha (TNF-α) are key soluble mediators that orchestrate inflammatory processes (11). Chemokines (e.g., CCL2, CXCL8) act as chemoattractants to recruit immune cells to sites of inflammation (12). Acute phase proteins (e.g., C-reactive protein) increase in response to inflammatory stimuli. Proteases (e.g., matrix metalloproteinases) degrade extracellular matrix components during tissue remodeling. Cellular markers like soluble adhesion molecules shed from activated leukocytes also serve as circulating inflammatory indicators (13).

Circulating levels of these markers can change dramatically during inflammatory conditions and correlate with disease activity, making them promising biomarker candidates. Moreover, many inflammatory mediators directly contribute to pathogenesis by promoting processes like immune cell infiltration, cytokine amplification loops, and tissue destruction (14). Quantifying circulating inflammatory markers may not only enable monitoring of disease status but also provide insights into the specific inflammatory pathways dysregulated in different conditions (15). This information could guide the development of targeted immunomodulatory therapies tailored to an individual’s inflammatory profile.

Mendelian randomization (MR) analysis is a robust method that utilizes genetic variants as instrumental variables to establish causality. Since alleles are randomly allocated at meiosis, MR is less susceptible to confounding or reverse causation that distorts observational studies (16). Recent large-scale genome-wide association studies (GWAS) have identified numerous genetic loci associated with levels of diverse circulating inflammatory molecules (17). These genetic instruments can be leveraged in MR frameworks to probe the causal impacts of inflammatory markers on disease outcomes. However, MR analysis examining the effects of inflammation on sciatica has not previously been undertaken.

In this study, we performed a comprehensive MR study evaluating causal relationships between 91 distinct plasma inflammatory markers and the risk of developing sciatica. The genetic determinants of inflammatory mediators were acquired from a comprehensive genome-wide association study (GWAS) involving a population of more than 14,000 individuals. The data on sciatica and genetic information were obtained from European cohorts consisting of more than 480,000 individuals. By integrating these datasets, we assessed the causal influence of circulating inflammatory levels on sciatica susceptibility. Our analysis represents the most extensive investigation to date of inflammatory mediators in sciatica using a genetic approach to strengthen causal inference.

Delineating causal inflammatory factors in sciatica may highlight novel targets for future therapies to manage this disabling condition. More broadly, this work demonstrates the utility of harnessing genetic instrumental variables and GWAS resources to illuminate causal immunologic mechanisms in pain pathologies. Our study provides a foundation for elucidating the role of inflammation in sciatica, with implications for improving treatment and prevention. Findings could catalyze the development of targeted anti-inflammatory medications as more effective precision medicine strategies for managing this common neuropathic pain syndrome.

## 2 Materials and methods

### 2.1 Study design

Based on a two-sample MR Analysis, we evaluated the causal relationship between 91 circulating inflammatory markers and sciatica. MR Uses genetic variation to represent risk factors, so valid instrumental variables (IVs) in causal reasoning must satisfy three key assumptions: (1) genetic variation is directly associated with exposure; (2) genetic variation is not associated with possible confounders between exposure and outcome; and (3) genetic variation does not affect outcome through pathways other than exposure (18). This study used sciatica data from the OpenGWAS, which included 484,598 Europeans, including 4,549 cases and 480,049 as the control group.

### 2.2 Circulating inflammatory markers GWAS data sources

Aggregate GWAS statistics for each circulating inflammatory marker are publicly available from the GWAS catalog (registration numbers from GCST90274758 to GCST90274848). This is a large-scale GWAS study that used the Olink Target platform to conduct genome-wide protein quantitative trait loci (pQTL) analysis on 91 plasma proteins measured by 14,824 participants.

### 2.3 Selection of instrumental variables

Since genetic variation is directly related to exposure, the significance level of IVs for each circulating inflammatory marker was set at 1 × 10^−5^. To obtain instrumental variables (IVs) for independent sites, we used the “TwoSampleMR” packet data with a linkage unbalance (LD) threshold set to R^2^ < 0.001 and an aggregation distance of 10,000 kb (19). For sciatica, we adjusted the significance level to 5 × 10^−6^, which is commonly used to represent genome-wide significance in GWAS, with an LD threshold of R^2^ < 0.001 and an aggregation distance of 10,000 kb.

### 2.4 Statistical analysis

**Figure 1:**
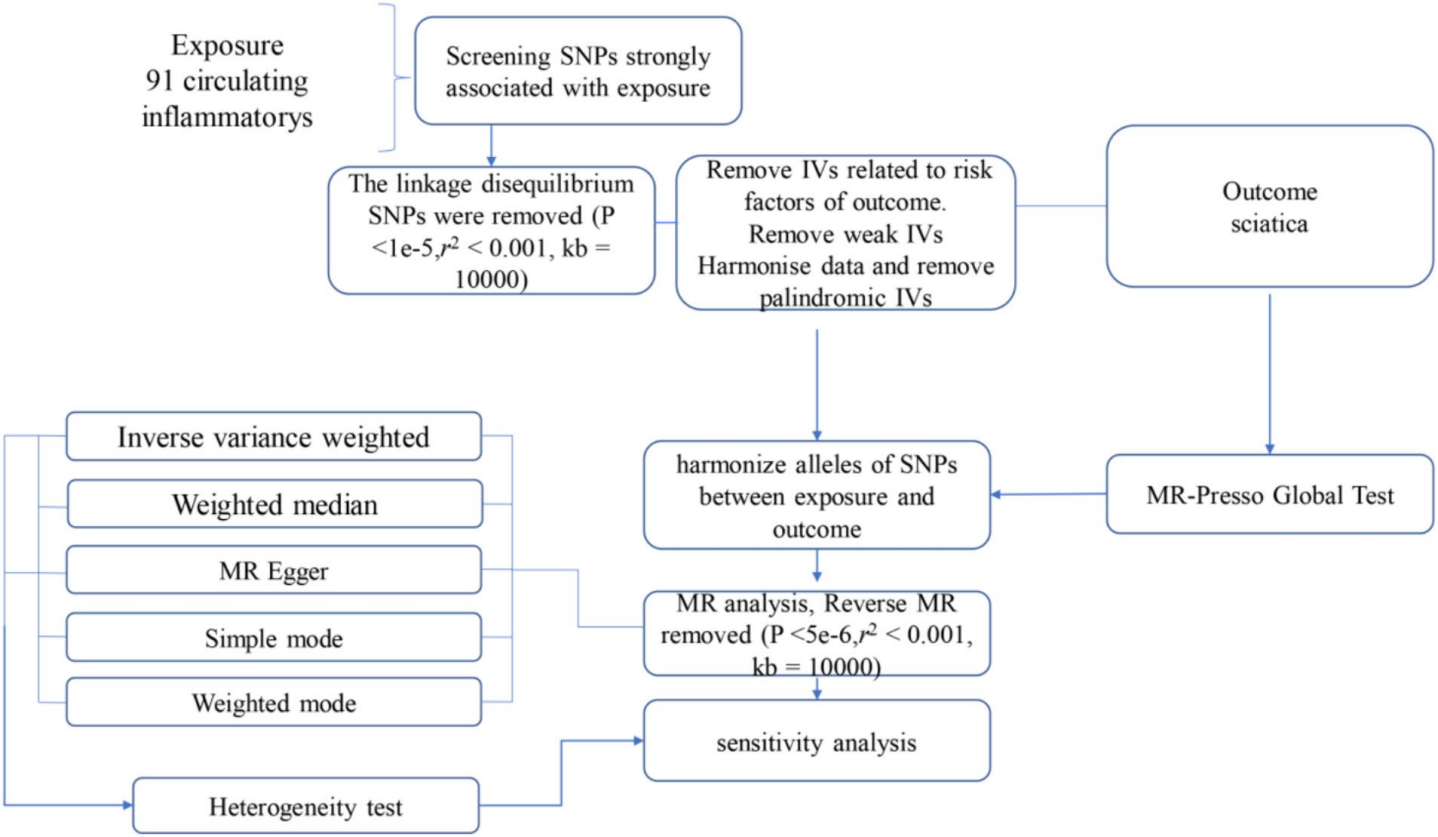
Flow diagram for quality control of the instrumental variables (IVs) and the entire Mendelian Randomization (MR) analysis process. SNPs, single-nucleotide polymorphisms; IVW, inverse variance weighted; MR, Mendelian Randomization; MR Presso, Mendelian Randomization Pleiotropy RESidual Sum, and Outlier.

The statistical analysis portion of our study, which investigated the causative impact of circulating inflammatory markers on the incidence of sciatica, was performed using R software, specifically version 4.2.1. R is widely used platform for statistical computing and graphics, accessible at (20).^1^ To ascertain the causal relationships between the 1,400 circulating inflammatory markers and sciatica, we primarily employed methods including inverse variance weighting (IVW), and weighted median-based estimation. These analyses were facilitated by the “TwoSampleMR” package, version 0.5.7, within the R software environment. This package is specifically designed for conducting MR analyses, providing tools for estimation, testing, and sensitivity analysis of causal effects (21). The IVW method is a standard approach in MR that combines the Wald estimates (ratio of the SNP-outcome association to the SNP-exposure association) from multiple genetic variants, weighting by the inverse variance of each SNP-outcome association. The weighted median and mode-based methods serve as supplementary approaches that provide robust causal estimates even when some of the instrumental variables are invalid if certain assumptions are met. These analyses were backed up by rigorous sensitivity analyses, including Cochran’s Q test to examine heterogeneity among the instrumental variables. Such thorough statistical evaluation ensures that the findings regarding the relationship between circulating inflammatory markers and sciatica are as dependable and accurate as possible given the data (22). The full process is shown in Figure 1.

## 3 Results

### 3.1 Exploration of the causal effect of circulating inflammatory markers on sciatica risk

**Figure 2:**
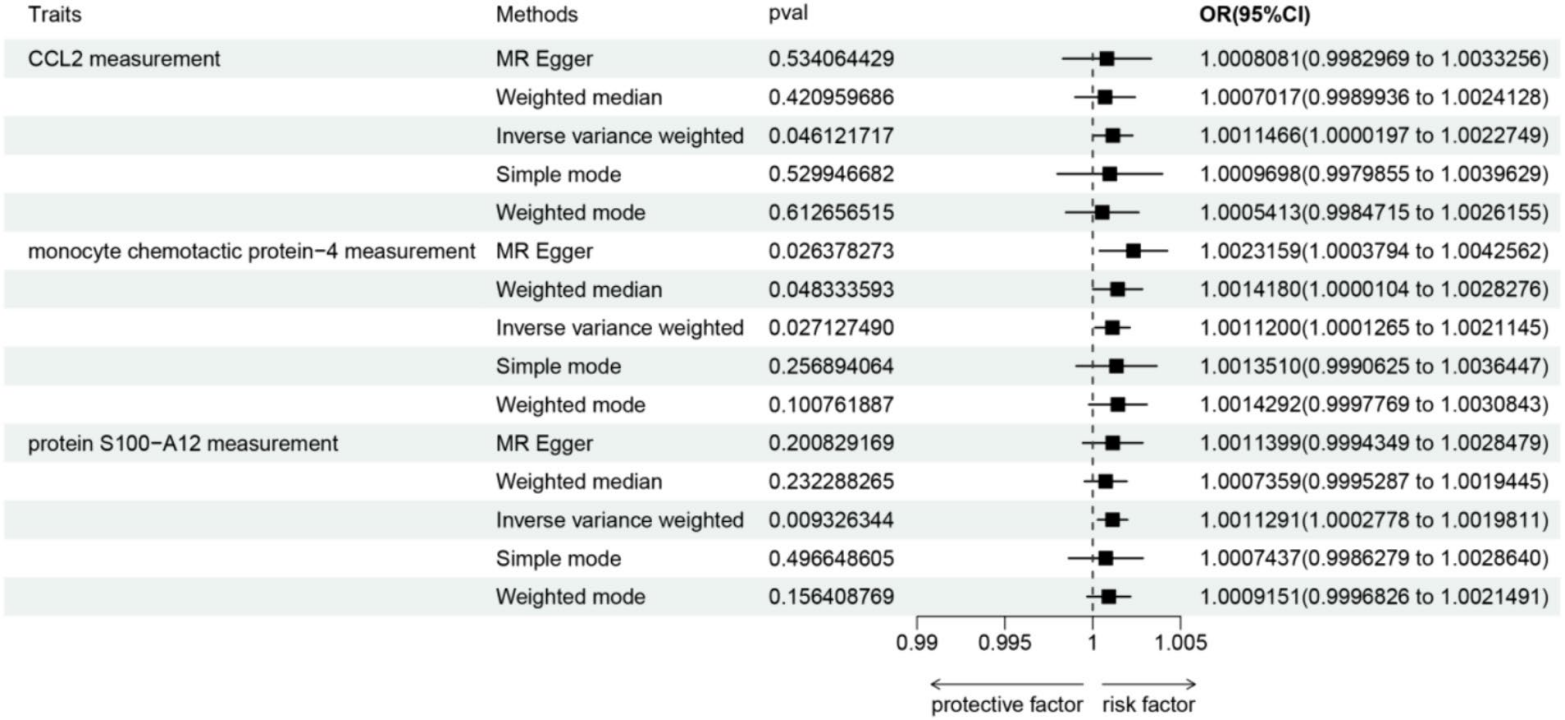
Forest plots depicting the causal associations between sciatica and circulating inflammatory markers. IVW, inverse variance weighting; CI, confidence interval.

At the significance level of 0.05, a total of 52 circulating inflammatorys were identified as causally associated with the development of sciatica. The CCL2 measurement (*p* = 0.046, OR = 1.00115, 95% CI = 1.00002–1.00227), monocyte chemotactic protein-4 measurement (*p* = 0.027, OR = 1.00112, 95% CI = 1.00013–1.00211), protein S100-A12 measurement (*p* = 0.009, OR = 1.00113, 95% CI =1.00028–1.00198) are positively correlated with sciatica (as shown in Figure 2). Results from sensitivity analyses demonstrate the robustness of the observed causal association (Supplementary Figure 1). Scatter plot and funnel plot also show the stability of the results (Supplementary Figures 2, 3). In addition, IVW results showed no heterogeneity (*p* > 0.05), and MR-egger and MR-presso results showed no horizontal pleiotropy (*p* > 0.05; Supplementary Table 1).

### 3.2 Exploration of the causal effect of sciatica risk on circulating inflammatory markers

To investigate the causal relationship between sciatica and circulating inflammatory markers, a two-sample Mendelian randomization (MR) analysis was employed, with the Inverse Variance Weighting (IVW) method as the primary analytical approach and other methods serving as supplementary. Subsequently, reverse MR was used to explore the impact of sciatica onset on the circulating inflammatory markers (as shown in Supplementary Table 2). The results indicate a positive correlation between sciatica and Fms-related tyrosine kinase 3 ligand levels (*p* = 0.033, OR = 12,146, 95% CI =2.1407–68,913,392). Conversely, there is a negative correlation between sciatica and Interleukin-1-alpha levels (*p* = 0.029, OR = 1.67E−05, 95% CI =8.79E−10–0.318), Interleukin-20 levels (*p* = 0.007, OR = 1.59E−06, 95% CI =8.95E−11–0.028), and Interleukin-33 levels (*p* = 0.029, OR = 7.62E−06, 95% CI =1.86E−10–0.312). Results from sensitivity analyses demonstrate the robustness of the observed causal association (Supplementary Figure 4). Scatter plots and funnel plots also show the stability of the results (Supplementary Figures 5, 6).

## 4 Discussion

In this study, we performed a comprehensive Mendelian randomization analysis evaluating causal associations between 91 circulating inflammatory markers and susceptibility to developing sciatica. Our findings provide robust genetic evidence that inflammatory pathways play an active role in driving sciatic nerve injury and pain. Multiple pro-inflammatory cytokines, chemokines, growth factors, and proteases demonstrated significant causal impacts on sciatica risk. The results nominate specific inflammatory molecules that may represent viable targets for future therapies to prevent or treat sciatica. More broadly, this study highlights the promise of leveraging genetic instrumental variables to elucidate causal immunologic mechanisms underlying neuropathic pain.

Overall, we identified over 50 inflammatory mediators that exhibited significant causal relationships with sciatica risk. The implicated molecules span a range of functions, from attracting immune cells to areas of tissue damage to directly inducing nociceptor sensitization and pain signaling (23). For instance, several key chemokines including CCL2, CCL11, CXCL5, CXCL6, and IL-16 were positively associated with sciatica. These chemotactic proteins contribute to pathogenesis by promoting immune cell infiltration and neuroinflammation within the sciatic nerve. We also found causal effects for proteases like MMP1, MMP10, and ADAMTS13 that can directly degrade extracellular matrix components of the nerve. Resultant nerve injury may trigger cascades of inflammation and neuroplastic changes underlying chronic sciatic pain (24, 25).

Additionally, our analysis revealed causal effects of pronociceptive signaling molecules like S100A12, an endogenous ligand for the RAGE pain receptor. Another notable finding was the positive causal association between Fms-related tyrosine kinase 3 ligand (FLT3L) and sciatica. FLT3L stimulates dendritic cell activity, which are specialized antigen presenting cells that bridge innate and adaptive immunity (26). Dendritic cell accumulations in damaged peripheral nerves can critically orchestrate pathological neuroimmune responses. Our study newly implicates this dendritic cell growth factor as a causal driver of sciatica, in addition to the chemokines that promote the recruitment of dendritic cells.

Conversely, a few anti-inflammatory molecules like IL-4 exhibited protective effects against sciatica development, further supporting the pathogenic role of inflammation. The breadth of implicated inflammatory mediators highlights the complex immunologic processes involved in sciatic nerve injury. Interestingly, our reverse MR analysis suggested potential bidirectional relationships between inflammation and sciatica. Sciatica caused subsequent elevations in FLT3L levels. This may reflect ongoing dendritic cell activation sustaining chronic inflammation and pain. Our integrated analysis provides temporal insights into inflammatory pathway activation over the course of sciatica.

These findings have several important clinical implications. First, the results identify novel therapeutic targets for more effective sciatica treatment. Current medications like NSAIDs lack specificity and have inadequate efficacy in many patients (8). The causal inflammatory mediators we identified could be selectively inhibited to ameliorate immune-driven pathologies without broad immunosuppression. For example, CCL2 blockade is being explored for neuropathic pain, while FLT3L inhibition may help dampen dendritic cell-mediated neuroinflammation. Targeting upstream initiators like FLT3L could potentially exert broader effects by suppressing multiple downstream components like chemokines and matrix proteases (27, 28).

Second, the implicated molecules could be evaluated as prognostic biomarkers to enable personalized management. Inflammatory marker panels could help stratify sciatica subgroups, predict treatment responses, or identify individuals requiring more aggressive interventions (29). Third, the findings support potential preventive approaches by controlling chronic inflammation earlier in high-risk groups. For instance, diet and lifestyle changes to reduce systemic inflammation may mitigate nerve injury risks (30). Overall, directly targeting causal inflammatory networks could offer synergistic benefits in both preventing and treating established sciatica.

Beyond sciatica, this work broadly demonstrates the utility of MR methods for unraveling the immunopathogenesis of neuropathic pain. Our analytical approach could be applied to illuminate inflammatory drivers of conditions like carpal tunnel syndrome, diabetic neuropathy, and radiculopathies using available GWAS resources. Insights from human genetic studies can complement animal models in guiding the translation of immunomodulatory pain therapies. Furthermore, the MR framework enables sequential mapping of inflammatory cascades from initiating cytokines to downstream mediators (16). Defining critical nodes across multi-step inflammatory pathways may reveal optimal intervention points for blocking pain.

The findings from this study have significant potential to improve the treatment landscape for debilitating sciatica. Currently, treatment options are limited, with medications like NSAIDs and opioid analgesics providing inadequate pain relief for many patients while carrying risks of adverse effects (31). The inflammatory mediators identified as causally linked to sciatica represent promising novel therapeutic targets. Selective inhibitors could be developed against upstream initiators like FLT3L to broadly disrupt pathogenic neuroinflammatory cascades (32). Alternatively, blocking downstream effectors like CCL2 or S100A12 could attenuate specific deleterious mechanisms like immune cell infiltration or nociceptor sensitization (33).

Importantly, targeting these causal inflammatory drivers could yield more efficacious and disease-modifying effects compared to conventional symptomatic treatments. Suppressing the root immunologic triggers may not only alleviate acute pain but also prevent permanent nerve damage and the transition to chronic, intractable neuropathic pain (34). Combination approaches inhibiting multiple nodes across inflammatory networks could provide synergistic benefits. Overall, the identification of key inflammatory pathways causally implicated in sciatica illuminates a new paradigm for developing targeted, mechanism-based therapies with greater potential for achieving meaningful pain relief and functional recovery in sciatica patients refractory to current management approaches.

Previous observational studies have reported associations between elevated levels of certain inflammatory markers like TNF-α, IL-6, and IL-8 in the serum or cerebrospinal fluid of sciatica patients compared to healthy controls (35). However, these studies could not establish causality due to potential confounding factors and reverse causality. Our MR analysis now provides robust genetic evidence confirming causal effects of multiple inflammatory mediators, including some previously implicated markers as well as several novel factors, on sciatica risk (36). The breadth of molecules spanning chemokines, proteases, growth factors, and signaling proteins highlights the complex neuroimmune mechanisms underlying sciatica pathogenesis.

Interestingly, many of the inflammatory molecules we identified as causally related to sciatica have also been implicated in other neuropathic pain disorders. For example, CCL2, S100A12, and matrix metalloproteinases are elevated in conditions like diabetic neuropathy, trigeminal neuralgia, and compressive radiculopathies (37). This suggests common inflammatory pathways may contribute to neuroinflammation and neuropathic pain across diverse etiologies. The causal inflammatory factors highlighted in our sciatica study could represent attractive therapeutic targets for managing a broader spectrum of neuropathic pain syndromes (38). Future studies directly investigating the effects of inhibiting these factors in preclinical models of nerve injury and clinical trials will be valuable for translating these findings.

It is important to recognize several limits. The predominantly European ancestry of the GWAS datasets warrants caution in generalizing conclusions. Further investigations involving larger and more diverse groups are required to validate the findings (39). Additionally, we only examined circulating inflammatory markers available in the GWAS dataset, providing an incomplete picture of all potential immunologic factors. Moving forward, integrating MR with multiple omics modalities will enable a more comprehensive mapping of neuroinflammation. Our findings require experimental validation to confirm the impacts of prioritized molecules on sciatic pathologies. Nevertheless, this work significantly advances the understanding of inflammatory mechanisms in sciatica.

## 5 Conclusion

In summary, our MR study provides convincing evidence that multiple inflammatory pathways causally contribute to sciatica pathogenesis. The findings illuminate underlying inflammatory cascades that may be targeted for improving the prevention and treatment of this common neuropathic pain disorder. This work establishes the basis for developing inflammation-centered prognostic biomarkers, preventive strategies, and directed therapies that have the potential to significantly improve the management of debilitating sciatica.

## Data availability statement

The original contributions presented in the study are included in the article/Supplementary material, further inquiries can be directed to the corresponding author.

## Ethics statement

The data for this study were obtained exclusively from publicly accessible databases containing anonymized participant information. As the dataset was pre-anonymized and publicly available, this study did not directly involve human participants and therefore did not require traditional ethical review. The design and use of data in this study followed all relevant data use guidelines and policies, ensuring sound and ethical use of data.

## Author contributions

YW: Writing – original draft, Writing – review & editing. YL: Software, Writing – review & editing. MZ: Software, Writing – review & editing. KH: Writing – review & editing. GT: Writing – original draft, Writing – review & editing.

## Footnotes

^1^ https://www.r-project.org/
