# Supplementary figures and images for: Causal association between circulating inflammatory markers and sciatica development: a Mendelian randomization study
Source: Front Neurol. 2024 Jul 2;15:1380719. doi: 10.3389/fneur.2024.1380719 (PMC11250389; doi:10.3389/fneur.2024.1380719)

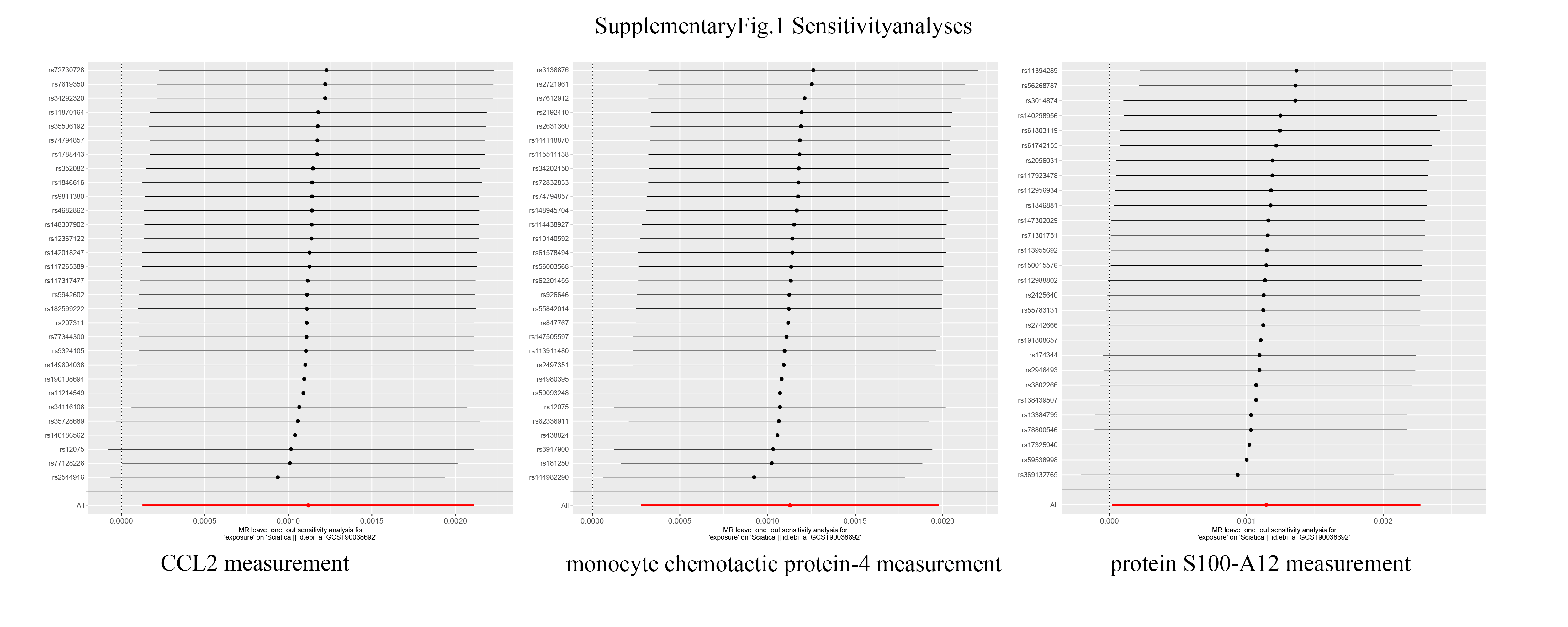

Supplement: Supplementary file 3 [file Image_1.TIF]

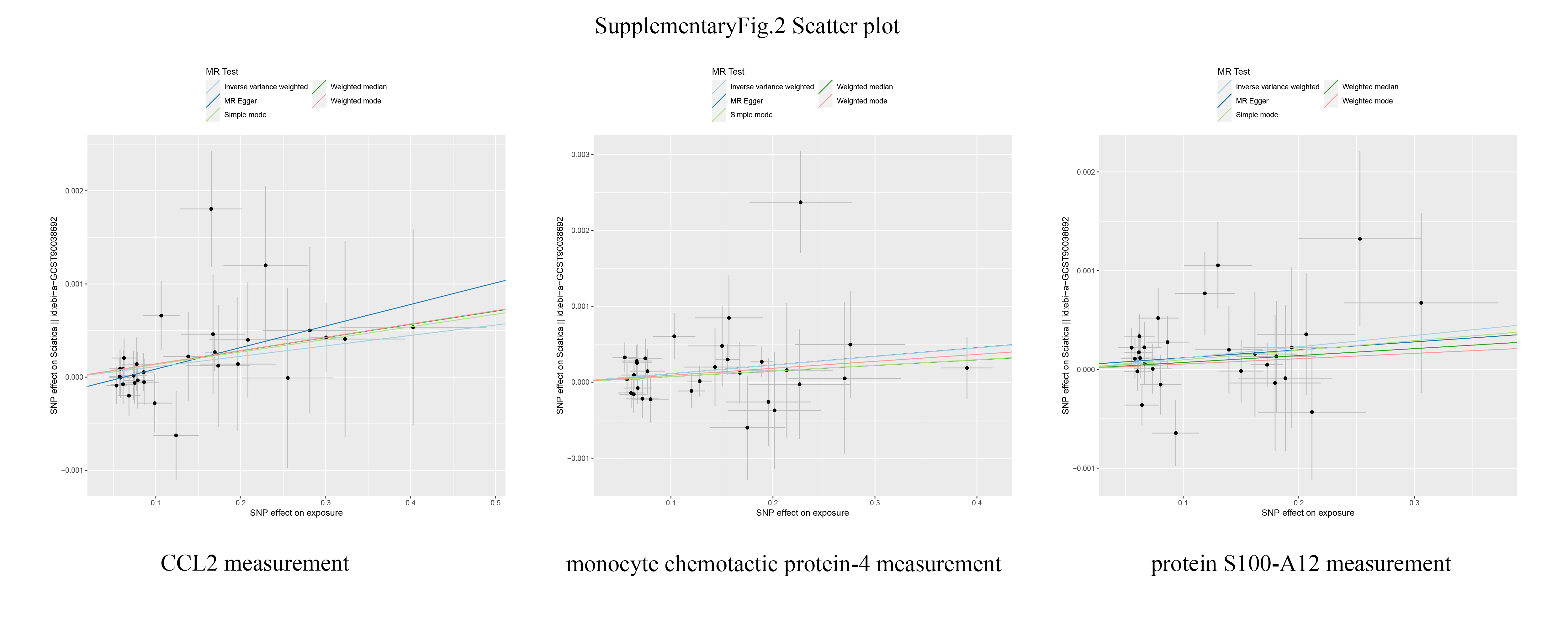

Supplement: Supplementary file 4 [file Image_2.TIF]

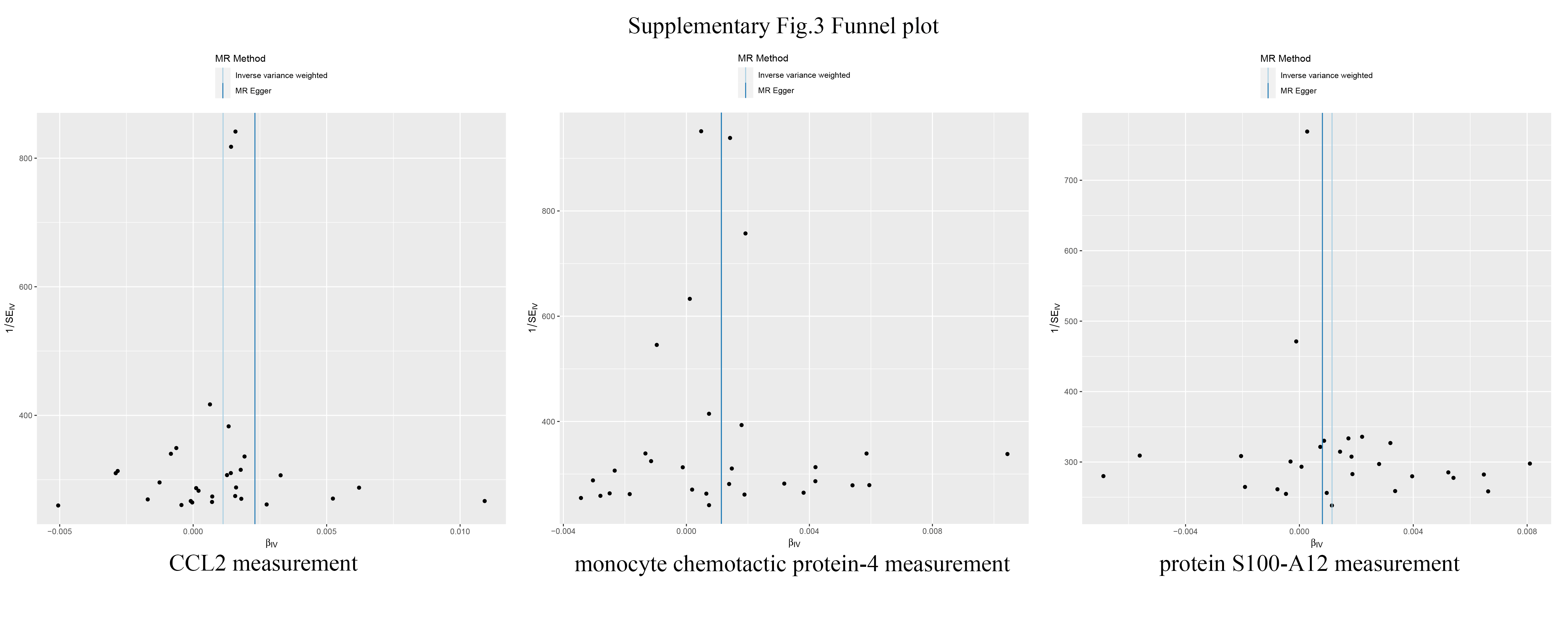

Supplement: Supplementary file 5 [file Image_3.TIF]

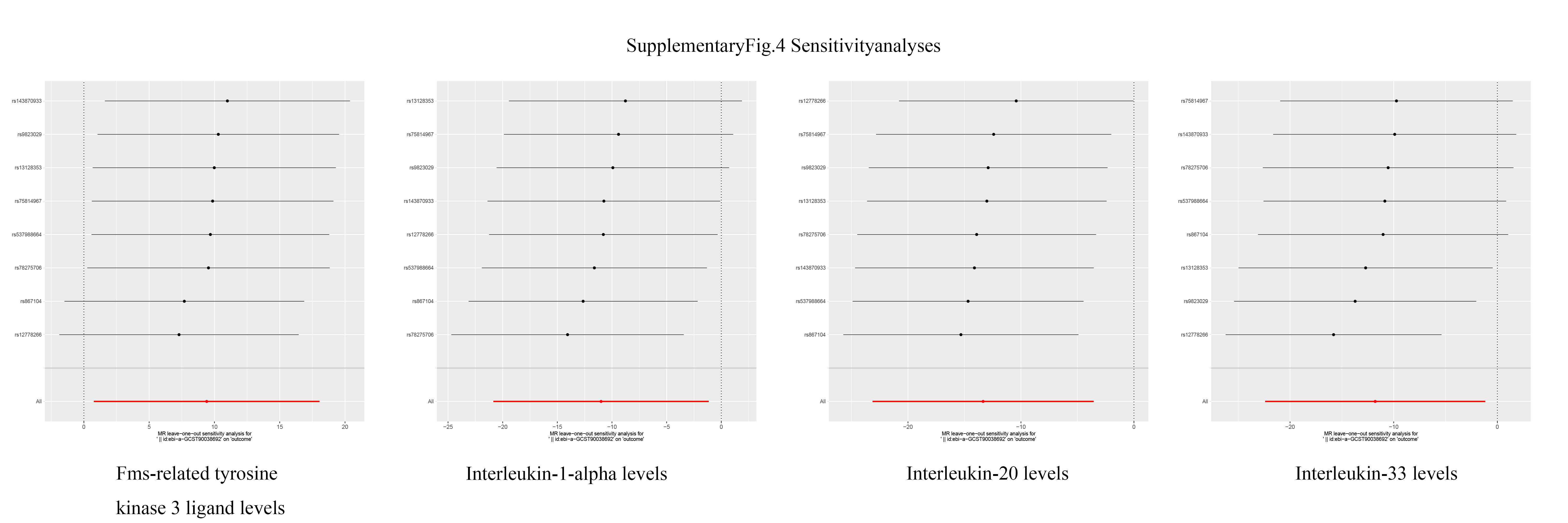

Supplement: Supplementary file 6 [file Image_4.TIF]

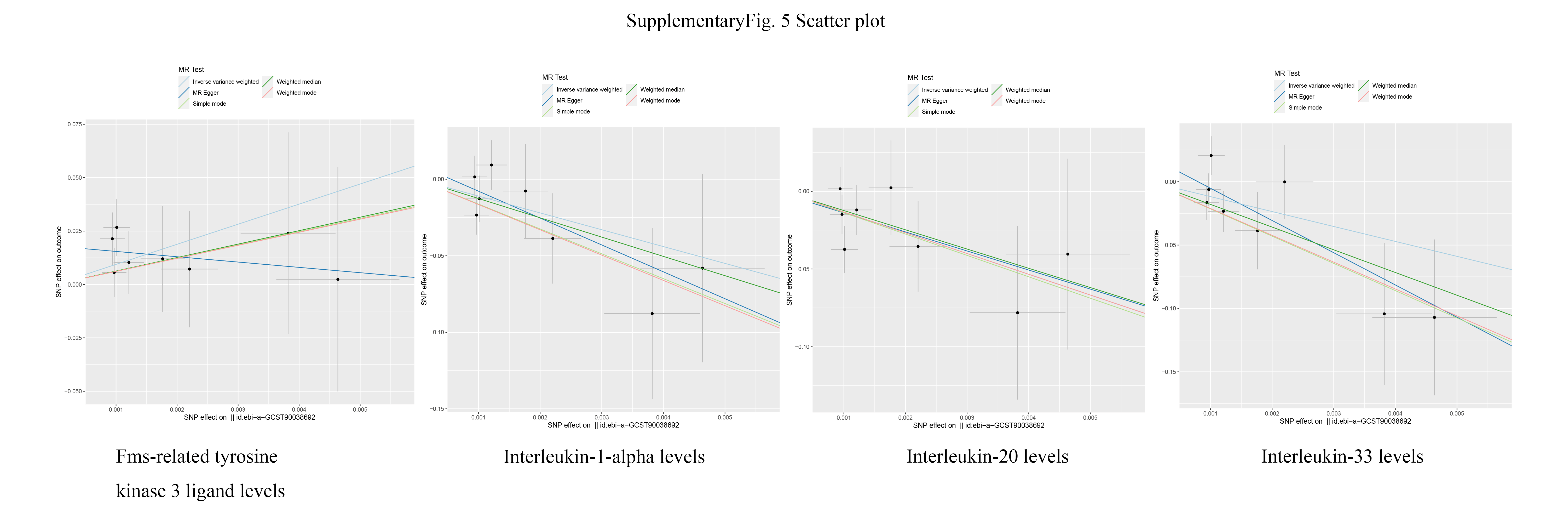

Supplement: Supplementary file 7 [file Image_5.TIF]

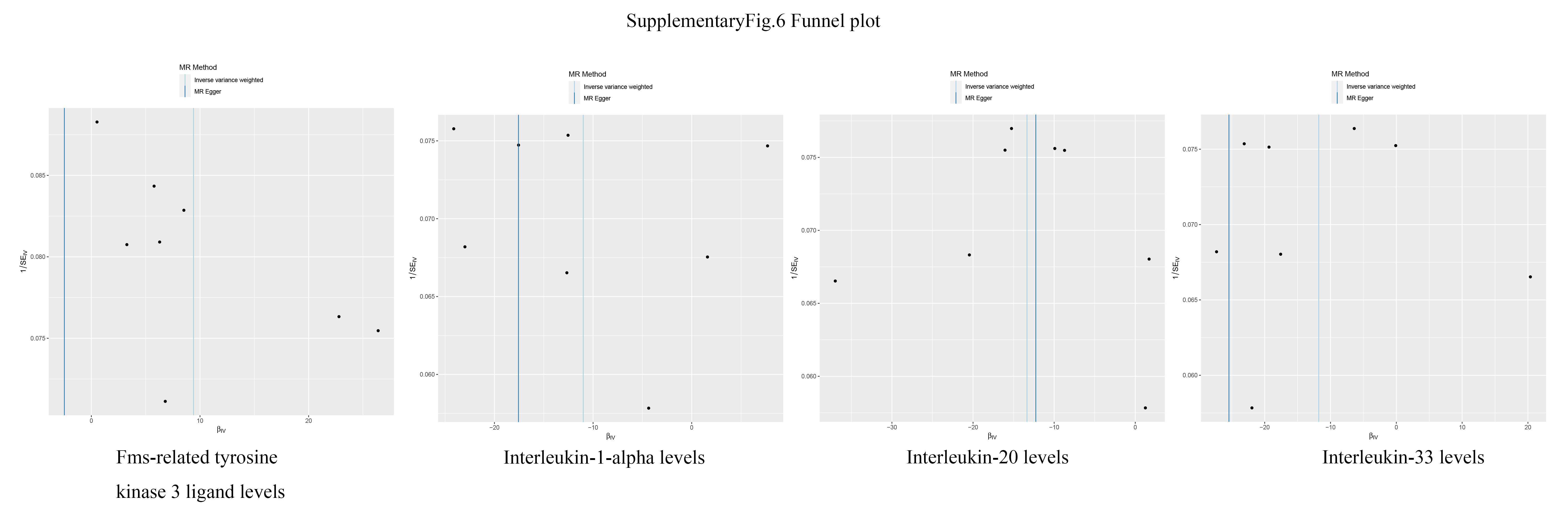

Supplement: Supplementary file 8 [file Image_6.TIF]
